# *LungGENIE*: the lung gene-expression and network imputation engine

**DOI:** 10.1186/s12864-025-11412-4

**Published:** 2025-03-10

**Authors:** Auyon J. Ghosh, Liam P. Coyne, Sanchit Panda, Aravind A. Menon, Matthew Moll, Michael A. Archer, Jason Wallen, Frank A. Middleton, Craig P. Hersh, Stephen J. Glatt, Jonathan L. Hess

**Affiliations:** 1 Division of Pulmonary, Critical Care, and Sleep Medicine, Department of Medicine, SUNY Upstate Medical University, 750 East Adams St, Syracuse, NY 13210 USA; 2 Department of Medicine, Johns Hopkins University School of Medicine, Baltimore, MD USA; 3 Division of Pulmonary, Critical Care, Allergy, and Sleep Medicine, Department of Medicine, Medical University of South Carolina, Charleston, SC USA; 4 Channing Division of Network Medicine, Brigham and Women’s Hospital, Boston, MA USA; 5 Division of Pulmonary and Critical Care Medicine, Brigham and Women’s Hospital, Boston, MA USA; 6 Harvard Medical School, Boston, MA USA; 7 Division of Thoracic Surgery, Department of Surgery, SUNY Upstate Medical University, Syracuse, NY USA; 8 Department of Neuroscience and Physiology, SUNY Upstate Medical University, Syracuse, NY USA; 9 Department of Psychiatry and Behavioral Sciences, SUNY Upstate Medical University, Syracuse, NY USA

**Keywords:** Gene-expression, Imputation, Chronic obstructive pulmonary disease

## Abstract

**Background:**

Few cohorts have study populations large enough to conduct molecular analysis of *ex vivo* lung tissue for genomic analyses. Transcriptome imputation is a non-invasive alternative with many potential applications. We present a novel transcriptome-imputation method called the Lung Gene Expression and Network Imputation Engine (*LungGENIE*) that uses principal components from blood gene-expression levels in a linear regression model to predict lung tissue-specific gene-expression.

**Methods:**

We use paired blood and lung RNA sequencing data from the Genotype-Tissue Expression (GTEx) project to train *LungGENIE* models. We replicate model performance in a unique dataset, where we generated RNA sequencing data from paired lung and blood samples available through the SUNY Upstate Biorepository (SUBR). We further demonstrate proof-of-concept application of *LungGENIE* models in an independent blood RNA sequencing data from the Genetic Epidemiology of COPD (COPDGene) study.

**Results:**

We show that *LungGENIE* prediction accuracies have higher correlation to measured lung tissue expression compared to existing *cis-*expression quantitative trait loci-based methods (median Pearson’s *r* = 0.25, IQR 0.19–0.32), with close to half of the reliably predicted transcripts being replicated in the testing dataset. Finally, we demonstrate significant correlation of differential expression results in chronic obstructive pulmonary disease (COPD) from imputed lung tissue gene-expression and differential expression results experimentally determined from lung tissue.

**Conclusion:**

Our results demonstrate that *LungGENIE* provides complementary results to existing expression quantitative trait loci-based methods and outperforms direct blood to lung results across internal cross-validation, external replication, and proof-of-concept in an independent dataset. Taken together, we establish *LungGENIE* as a tool with many potential applications in the study of lung diseases.

**Supplementary Information:**

The online version contains supplementary material available at 10.1186/s12864-025-11412-4.

## Introduction

Non-cancerous disorders of the lung, including chronic obstructive pulmonary disease (COPD), are associated with a significant global burden of morbidity and mortality. While molecular studies of human disease usually involve the primarily affected tissues, this is often not possible for non-cancerous lung disorders given the limited and vanishing indications for obtaining lung tissue in the clinical setting [1]. Thus, the availability of *exvivo* lung tissue for research purposes is similarly limited to relatively few existing study consortia and substantially smaller individual study cohorts [2, 3]. In contrast, collection of whole blood for molecular analysis, including RNA sequencing (RNAseq), from individuals with non-cancerous lung disorders is without significant risk [4, 5]. Several studies examining non-cancerous lung disorders have already generated transcriptomic data from blood in sample sizes that are orders of magnitude greater than those with data from lung tissue [6–9]. Transcriptome imputation of lung gene-expression from measures made in blood, therefore, offers an appealing and non-invasive alternative to obtaining lung tissue for direct transcriptome analysis.

Several existing software programs that estimate tissue-specific gene-expression, such as *TIGAR* and *PrediXcan*, leverage the effects of expression quantitative trait loci (eQTLs) to impute expression of syntenic genes (*cis*-eQTLs) [10, 11]. These methods have several strengths, including revealing putative mechanisms of single nucleotide polymorphisms (SNPs) identified by genome-wide association studies and moderate prediction of lung tissue-specific expression relative to other tissues. However, *PrediXcan* only predicted significant variance (i.e., *R*^*2*^ *≥* 0.01) in 7,400 genes in the lung, just a fraction of the lung transcriptome. In addition, *PrediXcan* is further limited to only static estimates of gene-expression and unable to predict temporal changes in lung tissue-specific gene-expression. Aside from the eQTL-based imputation methods, there are also cross-tissue transcriptome-imputation methods, including *TEEBoT* and *HYFA*, which have demonstrated impressive prediction of tissue-specific expression levels across many tissues [12, 13]. Following *TEEBot* and *HYFA*, there is growing interest in transcriptome imputation models that leverage peripherally accessible RNAs to predict ‘omic profiles for tissues not easily accessible in research participants or were collected in limited quantities by previous studies. However, each of these methods has important limitations in their development. For instance, *TEEBoT* models were trained on an earlier release of the Genotype-Tissue Expression (GTEx) Project that had significantly lower sample size than the current version 8 release of GTEx. *HYFA*, on the other hand, takes advantage of shared representations *via* transfer learning, enabling imputation profiles in ‘uncollected’ tissues based on gene expression profiles observed in peripheral blood and skin. Given the reliance on skin biopsies for *HYFA* models, this poses pragmatic challenges as skin biopsies are not routinely collected in the study of lung diseases. Furthermore, the increased invasiveness associated with obtaining skin biopsies poses challenges for practicality of *HYFA* models.

We therefore sought to enhance the existing methods and capitalize on the abundance of blood transcriptome data in existing study datasets to predict gene-expression in the lung solely based on observed peripheral gene-expression levels. We developed the Lung Gene-Expression and Network Imputation Engine (*LungGENIE*) using paired lung and blood transcriptomic data from GTEx Project. Similar to the Brain Gene-Expression and Network Imputation Engine (*BrainGENIE*), our previously established algorithm that uses gene-expression from blood to impute brain-regional gene-expression profiles, *LungGENIE* fills a critical gap in understanding lung molecular dynamics [14]. Notably, obtaining lung biopsies poses significant ethical and safety challenges akin to brain tissue; thus, these tissues are virtually off-limits in research participants [15, 16]. This limitation impedes our capacity to discover molecular signatures associated with pathological or therapeutic effects in the lungs of individuals living with chronic, non-cancerous lung diseases. Hence, *LungGENIE* enables profiling of lung-specific molecular profiles using minimally invasive strategies focusing on peripherally accessible RNA levels. Further, we demonstrated replication of the *LungGENIE* models in an independent dataset, using transcriptomic data generated from paired lung tissue and blood samples available through the SUNY Upstate Medical University Biorepository. Finally, we performed differential expression analyses using lung-tissue gene-expression levels imputed from blood RNAseq data from the Genetic Epidemiology of COPD (COPDGene) study and found significant overlap of predicted and directly measured differential expression in lung tissue in COPD.

## Methods

### Training and evaluation of *LungGENIE*

**Fig. 1:**
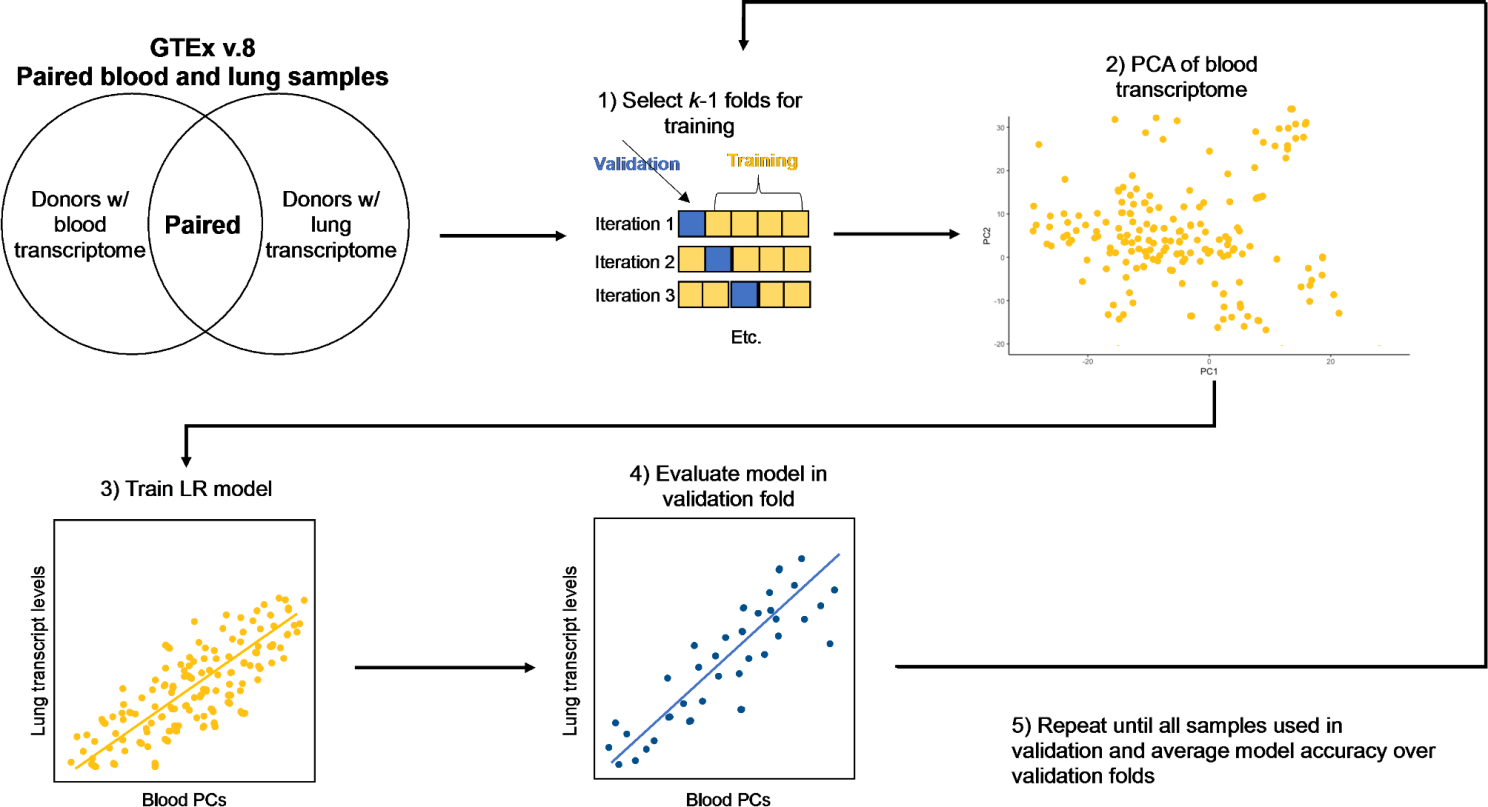
Protocol for training *LungGENIE* using paired blood and lung samples from GTEx

The approach used to train *LungGENIE* is illustrated schematically in Fig. 1.

We trained *LungGENIE* using paired lung and blood samples from GTEx v.8. *LungGENIE* uses principal components of transcriptome-wide gene expression data from blood to predict lung tissue-specific gene-expression levels. We used the coefficient of determination (*R*^*2*^) to measure the prediction accuracy of individual gene-expression levels from the observed gene-expression levels in the validation folds. We averaged *R*^*2*^ over 5 validation folds to estimate *LungGENIE* predictive performance.

Details regarding tissue collection, library preparation, sequencing, and normalization of GTEx data have been published previously [17]. We implemented five-fold cross-validation to estimate the internal predictive performance of *LungGENIE*. Paired lung tissue and blood transcriptome profiles from individuals in GTEx were assigned randomly to each fold. A principal component analysis (PCA) was performed on normalized blood transcriptome profiles. We then trained linear regression models to predict lung tissue-specific expression levels for individual genes using the top 20 principal components. The linear regression model used to train *LungGENIE* was constructed as: *Yi ~ β*_*0*_ *+ β*_*i*_*X*_*i*_*+…+ε*, where *Yi* denotes the level of expression of gene *i* in the lung, *β*_0_ denotes the intercept, and *β*_*i*_*X*_*i*_ denotes the estimated regression coefficient multiplied by the value of the *i*th principal component, and *ε* denotes the error term. The top 20 principal components method has been shown to have the best performance in *BrainGENIE*, compared to 5, 10, and 40 principal components. Similarly, prediction accuracies for linear regression were greater than or equal to elastic net regression. In addition, linear regression is computationally less intensive to train. We then deployed the trained models in the validation fold to estimate the predictive performance. We assessed the predictive performance with the coefficient of determination for observed and predicted per-gene expression levels (*R*^*2*^) in the validation fold. We repeated the process until each fold was used for validation, and then averaged the per-gene prediction over the five validation folds. We defined significantly predicted genes as those with a cross-validation (CV) *R*^*2*^ *≥* 0.01, adhering to the same criteria first outlined by *PrediXcan*, and Benjamini-Hochberg false-discovery rate-adjusted *p* value (FDR) < 0.05.

### Replication of *LungGENIE*

We sought to replicate the *LungGENIE* models in an independent external dataset generated from paired lung and blood samples from the SUNY Upstate Biorepository (SUBR). The samples were prospectively collected from individuals undergoing thoracic surgery for clinical indications to support future research studies. In the case of samples obtained during lung cancer surgery, we used samples that were the tissue farthest away from the margin. RNA was extracted from whole blood using the Monarch Total RNA Miniprep Kit (New England Biolabs). RNA was extracted from lung tissue using the miRNeasy Mini Kit (Qiagen). RNA quality and quantity was assessed with the RNA 6000 chip on the Agilent 2100 Bioanalyzer. RNA integrity score > 6 was used as the threshold of acceptable quality. Samples were included in subsequent analysis if they had > 10 million reads, > 80% of reads mapped, and *XIST* and Y chromosome expression matching reported sex. For sequencing library prep, RNA from blood and lung tissue samples was used as input to the Illumina Stranded Total RNA Prep with RiboZero Plus. Library size and quantity was assessed with the DNA 1000 chip on the Agilent 2100 Bioanalyzer. RNAseq data were generated on an Illumina NextSeq 2000 instrument, with a paired end 2 × 101 bp run. Results were saved in *FASTQ* format for analysis [18]. Reads were aligned to the GRCh38 genome using *TopHat2* and counts were generated using *Rsubread* with the Ensemble gtf [19]. Counts were normalized between samples using trimmed mean of M values (TMM).

As above, we used the coefficient of determination for observed and predicted per-gene expression levels (*R*^*2*^) as the metric for assessing prediction performance by comparing predicted vs. observed expression levels per transcript. Replicated models were identified by assessing transcripts that *LungGENIE* significantly predicted in GTEx *via* cross-validation (*R*² ≥ 0.01 and FDR ≤ 0.05). A two-tailed *z*-test was performed to compare the Pearson’s *r* correlation between GTEx cross-validation and the external SUNY Upstate Biorepository sample. Transcripts exhibiting no significant difference (uncorrected *p* > 0.05) in prediction accuracy between GTEx and SUBR, and demonstrating a Pearson’s *r* ≥ 0.1 in SUBR, were deemed replicated. Additionally, among transcripts demonstrating replicated prediction accuracy, we computed Pearson’s *r* correlations to measure the concordance of expression levels of transcripts across blood and lung tissue samples within the SUBR.

### Concordance with lung disease-related transcriptomic signatures

We aimed to assess whether *LungGENIE* can replicate transcriptomic changes associated with COPD in lung tissue. Toward this end, we deployed *LungGENIE* on independently collected *ex vivo* peripheral blood RNAseq data generated by the COPDGene study to impute lung-specific gene expression profiles for COPD cases and unaffected individuals without COPD (*n* individuals with COPD = 2,177, non-COPD comparison individuals = 1,783, total = 3,960). Details regarding sample collection and RNA sequencing in COPDGene have been previously published [4, 5, 8]. We performed standard pre-processing of raw gene counts as follows: remove genes with ≤ 1 count per million (CPM) in 100 or more participants, log_2_ transformation and quantile normalization of CPM values, and adjustment for batches using *ComBat* [20]. We excluded participants who were older than 70 years at the time of blood-sample collection, as this age range exceeded that of the individuals in GTEx who were used to train *LungGENIE*. Using the *LungGENIE*-generated lung-specific gene-expression data, we performed a differential expression analysis, with linear regression models that specified expression of each gene as a dependent variable and COPD disease status as the independent variable, to estimate group-mean differences in gene expression between COPD cases, defined as individuals with a ratio of forced expiration (FEV1) to forced vital capacity (FVC) less than 0.7, and non-COPD comparison participants, using the *R* package *limma*. Models were adjusted for sex, age, and percentage of four groups of circulating leukocytes (neutrophils, lymphocytes, monocytes, and eosinophils) taken from measured complete blood counts, as gene expression in blood can vary based on white blood cell proportions [21]. Corrections for multiple comparisons were made using the Benjamini-Hochberg false-discovery rate (FDR) procedure. We then calculated Pearson’s correlation coefficients of log_2_ fold changes after FDR correction to assess the similarity between the following sets of findings: (1) COPD-associated gene expression changes directly measured in lung tissue, reported by the Lung Tissue Research Consortium (LTRC) and (2) COPD-associated changes in gene expression found by *LungGENIE* [22]. This analysis sought to determine whether the transcriptome-wide picture of COPD in the lung—captured by the magnitude and direction differential expression across all measured transcripts—could be replicated in the blood-imputed lung transcriptome by *LungGENIE*. Additionally, we compared COPD-associated changes in gene expression from direct measurements in lung tissue with those observed in peripheral blood of COPD patients from the COPDGene study. This comparison allowed us to determine whether blood-imputed lung transcriptomes perform worse, as well as, or better than blood alone in capturing COPD-associated changes in gene expression found in lung tissue. Finally, we assessed the similarity of findings between COPD-associated different expression changes found in direct measurements of lung tissue with those imputed by *S-PrediXcan* from GWAS summary statistics obtained from the COPDGene study [23]. This comparison helped us to evaluate whether eQTL-predicted changes perform worse, as well as, or better than *LungGENIE* in recapitulating COPD-associated changes in gene expression found in lung tissue. We did not evaluate replication for any specific transcript in any of these abovementioned comparisons.

## Results

### Training of *LungGENIE* and comparison to *PrediXcan*

**Fig. 2:**
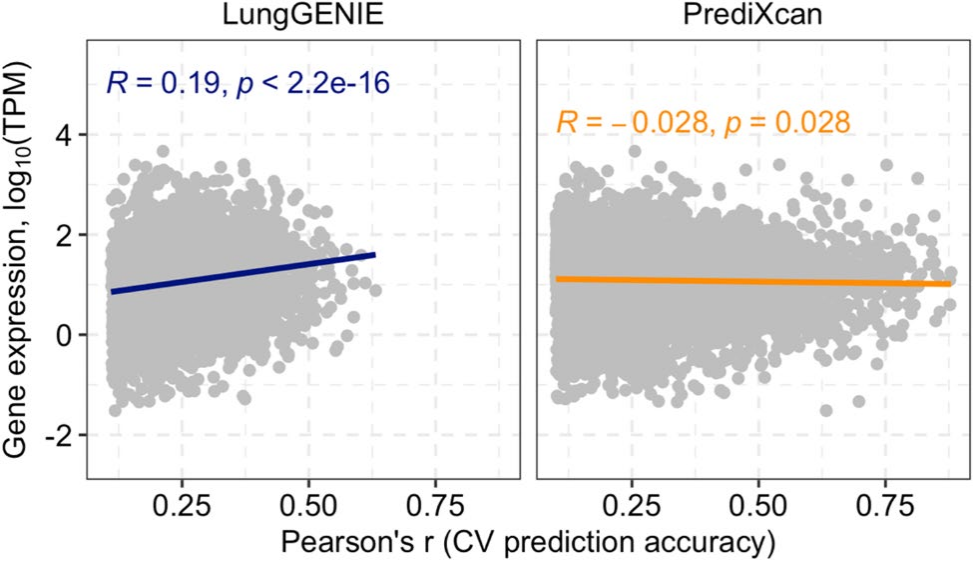
Comparison of *LungGENIE* to *PrediXcan*

We used paired blood and lung RNAseq data from 347 individuals from GTEx to train *LungGENIE*. We identified 19,304 genes that were significantly predicted by *LungGENIE* (*R*^*2*^ ≥ 0.01 and false-discovery rate adjusted *p*-value < 0.05). The mean cross-validation imputation accuracy for significantly imputed genes was Pearson’s *r* = 0.25, with an interquartile range of 0.19 to 0.32. We compared the cross-validation prediction accuracies between *LungGENIE* and *PrediXcan* relative to measured gene expression in the lung, restricted only to genes that were significantly predicted by both methods (Fig. 2).

Comparison of accuracies of gene-expression prediction between *LungGENIE* and *PrediXcan*. Side-by-side comparison of correlation of cross-validation predication accuracy and measured lung-tissue gene-expression between *LungGENIE* (left side) and *PrediXcan* (right side).

While *PrediXcan*-imputed cross-validation prediction accuracy was essentially uncorrelated to measured lung tissue gene expression, *LungGENIE* demonstrated a highly significant (but, on average, small) correlation of cross-validation prediction accuracy to lung tissue gene expression (Fig. 2).

### Replication of *LungGENIE*

We performed bulk RNAseq on paired lung-tissue and blood samples from 24 individuals (48 samples) from the SUBR. We excluded data from two individuals due to poor RNA quality (i.e., RIN < 6), resulting in 22 individuals and 44 samples included in subsequent analyses (Table 1). All samples were obtained from individuals with lung cancer, with adenocarcinoma as the predominant type (59%). The mean age in the cohort was 66.3 years and a majority of individuals were women (63.6%) and non-Hispanic white (90.9%). 54.5% of SUBR individuals were former smokers while 40.9% were current smokers (one individual was a never smoker). The mean smoking pack-years for the cohort was 28.4.

**Table 1:** Subject characteristics from SUBR

Characteristic	Overall
*N*	22
Age (years)	66.27 (8.4)
Male (%)	8 (36.4)
Non-Hispanic White (%)	20 (90.9)
Lung cancer subtype (%)	
Squamous	5 (22.7)
Adenocarcinoma	13 (59.1)
Carcinoid	1 (4.5)
Other	3 (13.6)
Smoking status (%)	
Current	9 (40.9)
Former	12 (54.5)
Never	1 (4.5)
Smoking pack-years	28.44 (15.3)

Data presented as mean (SD) or *n* (%) for continuous vs. categorical variables

**Fig. 3:**
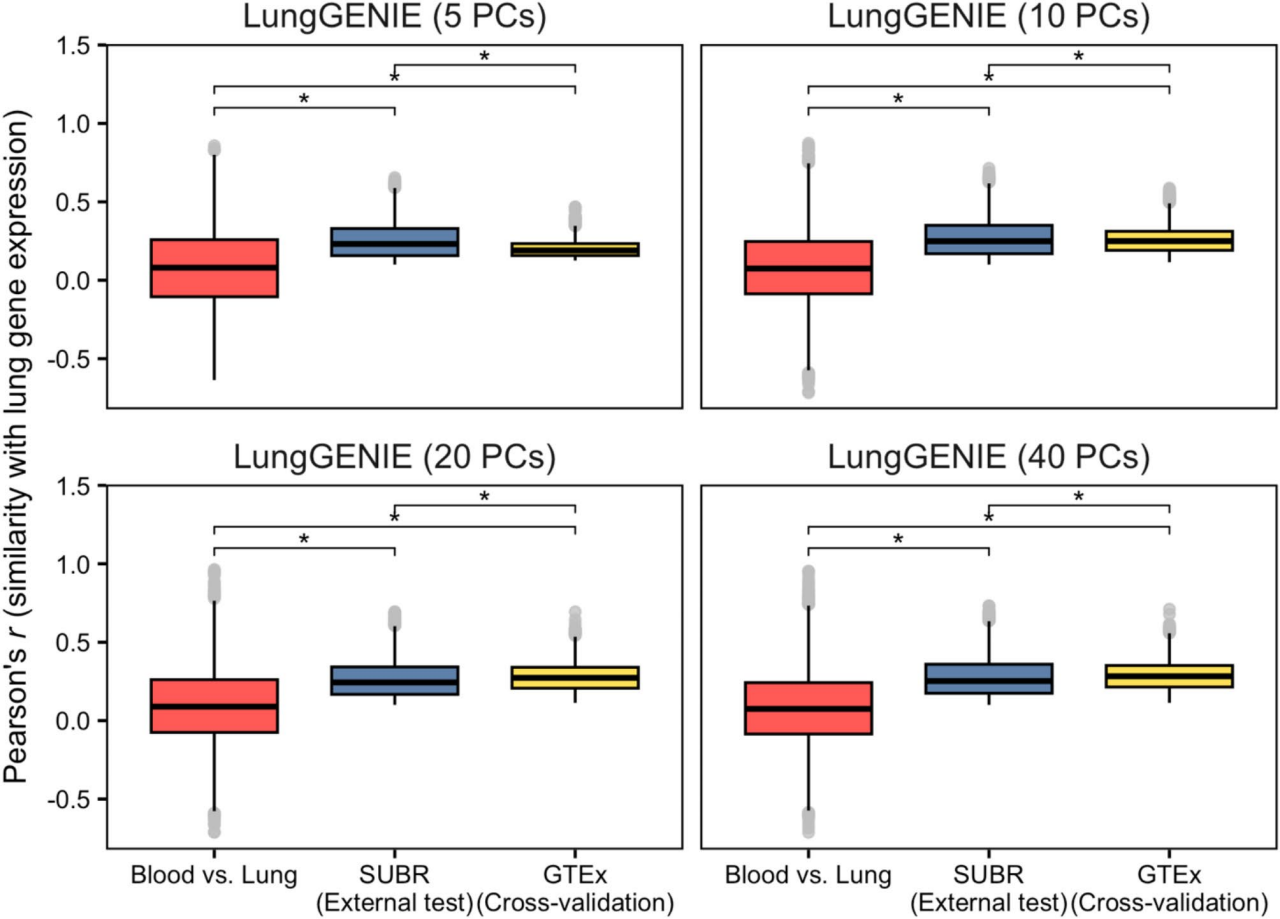
Comparison of correlation between imputed vs. observed gene expression in SUBR and GTEx across *LungGENIE* models Box-and-whisker plots showing the concordance between observed lung gene expression compared with *LungGENIE*-imputed transcripts and directly observed transcript levels in blood. The red and blue distributions depict the concordance of expression levels between blood vs. lung tissues, and between *LungGENIE*-imputed vs. lung tissues, respectively, within the SUBR dataset. These distributions specifically pertain to transcripts that demonstrated significant replication compared to cross-validation performance. The green distribution depicts the corresponding accuracy of *LungGENIE* derived from 5-fold cross-validation within GTEx. Asterisks (*) denote FDR-adjusted statistically significant differences in group means based on pairwise *t*-tests. Grey points depict transcripts that were beyond the interquartile range of each group.

We compared the replicability of *LungGENIE-*imputed vs. observed gene-expression levels across four models, varying by the number of principal components. We compared models by number of transcripts reliably imputed and mean cross-validation accuracy as well as number of replicated transcripts reliably imputed and mean validation accuracy. While the number of transcripts reliably imputed and mean CV accuracy improved with the number of principal components included in the *LungGENIE* model, the mean validation accuracy and number of replicated transcripts were highest with the *LungGENIE* model with 10 PCs (Table 2). We similarly compared correlation of *LungGENIE-*imputed gene expression by number of PCs with observed lung gene expression between SUNYBR, GTEx, and the correlation of blood vs. lung gene expression (Fig. 3).

**Table 2:** Overview of the replicability of prediction accuracy of imputed lung vs. observed lung expression levels for transcripts imputed by *LungGENIE* in tissue samples from the SUNY upstate biorepository (SUBR)

LungGENIE (# of PCs)	# of transcripts Imputed in SUBR	Mean CV accuracy	Mean validation accuracy	# replicated transcripts (%)
5	7,971	0.20	0.013	2,713 (34%)
10	12,875	0.25	0.090	6,324 (49%)
20	13,717	0.27	0.065	6,021 (44%)
40	14,038	0.29	0.057	6,227 (44%)

**Abbreviations**: Principal components (PCs), cross-validation (CV)

Correlation between imputed gene expression and observed gene expression was significant for all *LungGENIE* models in both SUNYBR and GTEx samples, compared to the correlation of blood vs. lung gene expression.

### Concordance with lung disease-related transcriptomic signatures

**Fig. 4:**
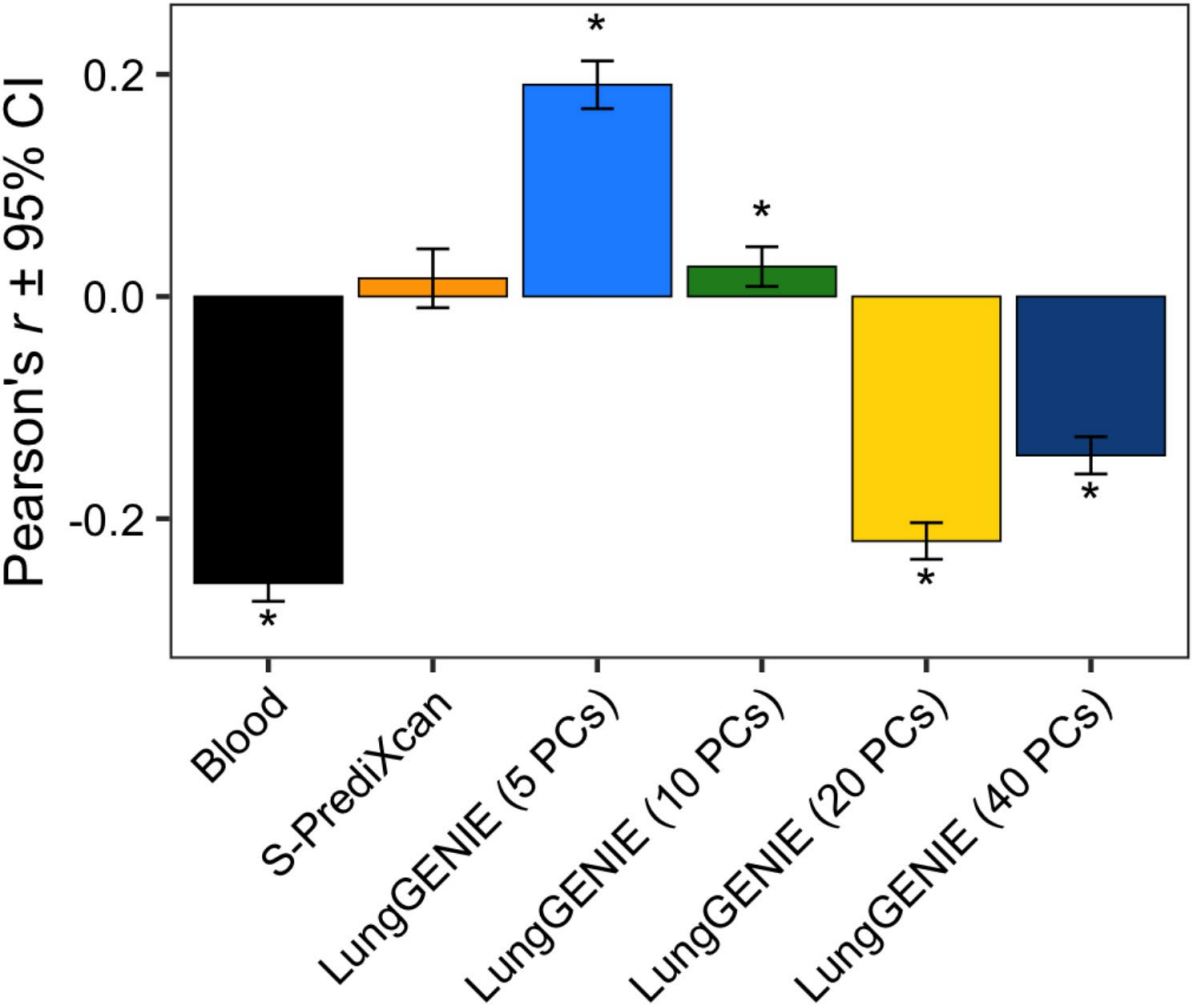
Concordance between COPD-related differential expression results from imputed lung gene expression with experimentally measured differential expression from lung tissue The concordance, measured by Pearson’s correlation coefficients, between COPD-related differential gene expression (DGE) log_2_ fold changes detected in peripheral blood and those inferred by *LungGENIE* and *S-PrediXcan*, was compared to DGE signals derived from lung tissue RNAseq from the Lung Tissue Research Consortium. Asterisks (*) depict correlations that reached a statistical significance threshold of FDR *p* < 0.05.

Due to the age range of study participants from the training dataset (GTEx), we excluded samples from individuals over the age of 70 from our analyses in COPDGene. We therefore retained 2,694 individuals in our downstream analysis, including 911 individuals with COPD. We imputed lung gene-expression using *LungGENIE* models with 5, 10, 20, and 40 PCs, as well as using *S-PrediXcan*. After performing differential-expression analysis between individuals with COPD and control individuals, and correcting for multiple-testing, we compared correlation of differential-expression results (log_2_ fold changes) associated with COPD to our previously published experimentally measured differential-expression results in lung tissue from LTRC between individuals with COPD and control individuals (Fig. 4).

We also included the correlation of differential-expression results from blood gene expression as an additional comparison. The *LungGENIE* model with 5 PCs had the highest positive and significant correlation, followed sequentially by the *LungGENIE* model with 10 PCs and *S-PrediXcan*. Conversely, the differential expression results from imputed lung gene expression from *LungGENIE* models with 20 and 40 PCs both had negative correlation with the measured differential expression results, similar to the correlation with the differential expression results from blood.

## Discussion

In the present study, we introduce a computational method, called *LungGENIE*, that can be used to predict lung gene-expression levels using gene expression from peripheral blood. We further show that, across internal cross-validation, external replication, and proof-of-concept demonstration in an independent dataset, *LungGENIE* outperforms direct comparison of blood to lung results, and may augment results from existing gene-expression imputation methods, including *PrediXcan*. Specifically, we find that, for concordance with COPD differential expression results measured from lung tissue, the *LungGENIE* model with 5 PCs had the best performance.

The quest for developing non-invasive methods of estimating tissue-specific gene expression is ongoing, and particularly relevant for advancing the study of chronic, non-cancerous lung diseases. For example, the question of obtaining lung tissue for aid in diagnosis of interstitial lung diseases (ILD) remains fraught [24]. The most recent international society guidelines give no recommendation, for or against, the use of a genomic classifier, generated using whole transcriptome RNAseq data from lung/bronchial tissue obtained *via* transbronchial biopsy to elucidate the diagnosis of IPF in ILDs of unknown type [1]. Part of the reason for the committee’s hesitancy was due to the concerns inherent to performing transbronchial biopsies, which have up to a 30% complication rate [25, 26]. *LungGENIE*, therefore, has the potential to bridge this important gap by obviating the need for lung tissue by imputing the data necessary to employ the genomic classifier and help clarify the diagnosis in undifferentiated ILDs.

In contrast, the diagnosis of COPD is made through an assessment of lung physiology with spirometry, and an emerging role of qualitative and quantitative chest CT imaging, without any role for lung-tissue histopathologic or genomic analysis [27]. However, there is substantial clinical heterogeneity observed in COPD that suggests subtypes of disease with important pathobiological differences [28]. For instance, eosinophilic COPD has been shown to be an important treatable trait, where administration of a monoclonal antibody against the interleukin-4 receptor leads to a clinically relevant reduction in the rate of COPD exacerbations in individuals with COPD and a high level of circulating eosinophils [29, 30]. Other than eosinophilic COPD, however, there are few subtypes of COPD with clear differences in underlying pathobiology (i.e., endotypes) based on insights from the peripheral blood. On the other hand, we have previously shown that while individuals with COPD who are heterozygous for the *SERPINA1* Z allele have no differences in blood gene expression, there are subtle differences in lung-tissue gene expression that could be relevant to the observed clinical and radiographic differences [31]. Thus, broader application of *LungGENIE* on blood gene-expression data from individuals with COPD could be used to identify additional subtypes and potentially treatable traits.

There have been other tissue-specific gene expression tools that have been published in the last few years [12, 13]. Our method bears some similarities, including the use of GTEx as the primary dataset used to train our models as well as using the principal components of blood gene expression to estimate tissue-specific gene expression. However, our method stands out in several ways. First, the previous method leveraging PCA from blood gene expression, *TEEBoT*, used an older and smaller version of GTEx (v.6). *LungGENIE* models were trained using GTEx v.8, which includes a larger sample size (*TEEBoT* 217 lung samples, no independent replication vs. *LungGENIE* 347 lung samples, 24 lung samples in replication). This led to an increase in the number of imputed transcripts (*TEEBoT* 12,820 imputed transcripts vs. *LungGENIE* 14,038 imputed transcripts in the replication dataset). Second, we were able to integrate an independent dataset of paired lung and blood gene-expression data to externally replicate our models. To our knowledge, the paired lung and blood gene-expression data from the SUNY Upstate Biorepository represent the only existing dataset of its kind outside of GTEx. Existing datasets, including LTRC, have paired lung and blood samples but only have gene-expression data from one tissue (in the case of LTRC, only lung tissue RNAseq data are available). The ability to demonstrate replication of the performance of our models in an independent dataset is a unique attribute of *LungGENIE* compared to all other lung tissue-specific gene-expression imputation tools. Third, by validating *LungGENIE* in blood gene-expression data from a large, national cohort enriched for individuals with COPD, we were able to show the immediate impact that *LungGENIE* can have on studies using transcriptomic data to better understanding chronic lung diseases.

Despite these strengths, we must acknowledge that our study has limitations. First, while *LungGENIE* models significantly predicted a majority of the transcriptome, a non-negligible portion of the transcriptome was not reliably imputed. This highlights the importance of *LungGENIE* as a tool to be used in parallel with other imputation tools to capture the entire transcriptome, or as much of it as possible. Future iterations of *LungGENIE* may include SNP data to better account for *cis*-eQTL effects, which may improve the portion of the transcriptome that is reliably imputed. Next, we were unable to incorporate longitudinal blood gene-expression, which can potentially be mapped onto states of risk, illness, treatment, and potentially recovery. The addition of serially collected gene-expression data may also improve prediction of transcriptomic states in the lung related to disease [9]. Although gene expression trajectories have been mapped onto eQTLs, these relationships remain unexplored in the context of the lung transcriptome. Until such insights are provided, predictions from transcriptome-imputation methods based on genetic data will merely reflect static gene expression profiles [32]. Finally, our replication dataset was an order of magnitude smaller than the training dataset, which limits our statistical power to detect reliably replicated predicted genes. We aim to continue to refine *LungGENIE* by incorporating additional paired lung and blood sequencing samples in our biorepository to expand our dataset and integrating additional novel methods, including artificial intelligence, single cell transcriptomics, and RNA sequencing from multiple time points.

*LungGENIE* has several strengths compared to methods with a similar goal and demonstrates these strengths across a unique combination of datasets. We intend *LungGENIE* to complement the existing array of genetic-based imputation methods. There are several potential future applications of *LungGENIE*, including modeling the response to environmental insults and potential new therapies as well as lung-related trajectories across multiple timepoints in an individual’s lifetime.

## Electronic supplementary material

Below is the link to the electronic supplementary material.

Supplementary Material 1

## Data Availability

Data from GTEx are publicly available. Data from SUBR will be available upon request following a one-year embargo period after publication. Data from COPDGene are available to the scientific committee through the Database of Genotypes and Phenotypes (dbGaP) through the National Center for Biotechnology Information.The source code and training data for LungGENIE can be accessed online (https://github.com/hessJ/LungGENIE).
