# Supplementary material for: LungGENIE: the lung gene-expression and network imputation engine
Source: BMC Genomics. 2025 Mar 10;26:227. doi: 10.1186/s12864-025-11412-4 (PMC11892309; doi:10.1186/s12864-025-11412-4)
Supplement: Supplementary file 1 — Supplementary Material 1 [file 12864_2025_11412_MOESM1_ESM.docx]

Online supplemental for:

*LungGENIE*: The Lung Gene-Expression and Network Imputation Enginge

Auyon J. Ghosh, Liam P. Coyne, Sanchit Panda, Aravind A. Menon, Matthew Moll, Michael A. Archer, Jason Wallen, Frank A. Middleton, Craig P. Hersh, Stephen J. Glatt, Jonathan L. Hess

**Methods:**

*Normalization of RNAseq data from GTEx*

Total RNA was extracted from whole blood stored in PAXgene Blood RNA (Qiaqen) tubes. Total RNA was extracted from frozen lung tissue. All RNA samples that were used for RNAseq had RNA integrity number (RIN) values > 6.0 as measured by Aligent Bioanalyzer. Additional details related to tissue collection, library preparation, and sequencing were previously described by the GTEx Consortium. Gene-level read counts were summarized based on the Gencode 26 (GRCh38) transcript model. We took the following steps to pre-process gene-counts for analysis, including: retain genes with >0.1 read per kilobase per million (RPKM) and >5 read counts in at least 10 donors, quantile-normalize RPKM to adjust for between-sample variation (using *limma*), and inverse-rank normalization.

*Normalization of RNAseq data from SUBR*

After alignment to GRCh38 using *Bowtie*, genes were filtered to remove any with <1 in >80% of samples, then TPM normalized with add 1 and log2 transformation.
